# Fusion of visible and infrared images using GE-WA model and VGG-19 network

**DOI:** 10.1038/s41598-023-27391-z

**Published:** 2023-01-05

**Authors:** Weiqiang Fan, Xiaoyu Li, Zhongchao Liu

**Affiliations:** 1 School of Electronic Information Engineering, Inner Mongolia University, Hohhot, 010021 China; 2 School of Mechanical Electronic and Information Engineering, China University of Mining and Technology-Beijing, Beijing, 100083 China; 3 School of Intelligent Manufacturing, Nanyang Institute of Technology, Nanyang, 473004 China

**Keywords:** Electrical and electronic engineering, Applied optics

## Abstract

For the low computational efficiency, the existence of false targets, blurred targets, and halo occluded targets of existing image fusion models, a novel fusion method of visible and infrared images using GE-WA model and VGG-19 network is proposed. First, Laplacian is used to decompose the visible and infrared images into basic images and detail content. Next, a Gaussian estimation function is constructed, and a basic fusion scheme using the GE-WA model is designed to obtain a basic fusion image that eliminates halo of visible image. Then, the pre-trained VGG-19 network and the multi-layer fusion strategy are used to extract the fusion of different depth features of the visible and infrared images, and also obtain the fused detail content with different depth features. Finally, the fusion image is reconstructed by the basic image and detail content after fusion. The experiments show that the comprehensive evaluation FQ of the proposed method is better than other comparison methods, and has better performance in the aspects of image fusion speed, halo elimination of visible image, and image fusion quality, which is more suitable for visible and infrared image fusion in complex environments.

## Introduction

Image fusion aims to process the images of the same target or scene collected by multi-source sensors through specific algorithms to obtain a more accurate, comprehensive and reliable image description of the target or scene interpretation, so that it can combine the spatial characteristics of different band images^1^. Infrared image is imaged by the thermal radiation characteristics of objects, which has strong anti-interference ability and is easy to capture thermal targets in dark environments or camouflage^2,3^, but it is not sensitive to brightness changes and lack scene details. Visible image is imaged using the irradiation-reflection characteristics of the object^4^, which has high resolution and rich scene details, but it is susceptible to interference from external factors. Therefore, the fusion of infrared and visible image can integrate salient features of the two^5^, reduce the redundant information of images, and improve the accuracy of scene analysis and target positioning in complex environments. At present, image fusion technology has been widely used in troubleshooting, military reconnaissance, smart transportation, security monitoring, medical diagnosis and other fields^6–9^.

In recent years, in order to improve the ability of image detection, recognition, and understanding, scholars at home and abroad have gradually started to study different spectral image fusion methods^10,11^. The main research methods include transform domain (TD)^12^, spatial domain (SD)^13^, sparse dictionary learning^14,15^, and data-driven^16,17^. Among them, the fusion strategies based on TD mainly include discrete wavelet transform (DWT)^18^, contourlet transform, curvelet transform, dual-tree complex wavelet transform (DTCWT), Laplacian pyramid transform (LPT), non-subsampled contourlet transform (NSCT)^19^, non-subsampled shearlet transform (NSST)^20^, non-subsampled dual-tree complex contourlet transform and other methods^21,22^. The fusion strategies based on the SD mainly include intensity hue saturation (IHS), YUV^23^, principal component analysis (PCA), independent component analysis (ICA)^24^, brovery transform^25^, gram-schmidt^26^, and the improvement method of the above method, etc. The fusion strategies based on data-driven mainly use different neural models, such as convolutional neural networks, recurrent neural networks, and generative adversarial networks, etc.^8,11^.

The fusion strategies based on the TD obtains the basic image and the detail content through the transform function, uses different fusion strategies to fuse its coefficients, and utilizes the inverse transform function to obtain the fused image^9,13^. Among them, DWT has multi-scale and local time–frequency characteristics, but DWT does not have offset invariance and multi-directionality, and cannot effectively express the texture and edge features of 2D images in various directions. Contourlet and Curvelet transform have the advantages of DWT, and overcome the shortcomings of insufficient number of DWT directions, but lack of offset invariance^27^, resulting in the fusion image prone to pseudo Gibbs phenomenon, affecting the visual effect of the image. DTCWT can significantly improve the offset sensitivity and direction selectivity of DWT, but DTCWT and DWT have similar fusion strategies and cannot effectively improve the defects of fusion images^8^. NSCT and NSST have all the advantages of the above TD methods and offset invariance^27^, but similar to other TD methods, the fused image is prone to ghosting in the background and the edge of the salient target^28^, and the image fusion process is time-consuming and low efficiency^21,27^. The fusion strategies based on the SD uses the region division method to select different regions, and uses different fusion rules to achieve the image fusion of different regions^21^. Among them, the pixel fusion method has high efficiency, and can better retain the area information of infrared target and the detailed features of visible target, but SD method only fuses the pixels, it is easy to lose part of the detailed information^8,9^. Sparse dictionary learning aims to learn an over-complete dictionary from a large number of high-quality images to obtain an effective sparse representation of the source image^15^, but the most of fusion methods are computationally complex and need to determine the sparse representation model and fusion rules based on prior knowledge, which has great limitations^8,16^. The fusion strategies based on data-driven uses different neural network models to extract deep features of the source images, and realize heterogeneous image fusion by feature selection and feature fusion^8,29^. The main advantages of data-driven are similar to CNN-based classification tasks, which remove the complicated process of manually setting parameters, and make it easier to obtain better fusion results. However, data-driven also has some disadvantages, such as large amount of computation, poor model generalization ability, and complex network design, etc.^9,11^.

The above-mentioned image fusion methods can’t meet application scenarios with high real-time requirements such as equipment fault diagnosis, night security, and fire monitoring, and have disadvantages such as complex fusion process, loss of detailed information, and poor anti-light interference ability. This paper proposes an image fusion method using GE-WA model and VGG-19 network. Firstly, the source image is decomposed into basic image and detail content by Laplacian operator. Secondly, a fusion model based on GE-WA is designed to realize the basic image fusion. Then, the multi-layer depth features of detail content are extracted by the pre-trained VGG-19 network, and feature maps are constructed using different depth features. Meanwhile, a weighted fusion and maximum selection strategy based on detail content is used to fuse the feature maps of different depths to obtain the fused detailed content. Finally, the fused basic image and detailed content are reconstructed.

In summary, the main contributions of this paper are the following three folders. (1) A fusion model based on GE-WA is designed to eliminate the halo of the visible basic image, improve the anti-light interference ability of the fusion method. (2) A weighted fusion strategy based on detail content is designed to fuse the same depth feature maps of visible and infrared detail content, and a maximum selection strategy is used to fuse the fusion feature maps of different depths to obtain the fusion detail content. (3) A novel fusion method of visible and infrared images using GE-WA model and VGG-19 network is proposed, which overcomes the shortcomings of traditional fusion methods such as low fusion efficiency and easy loss of detailed information, and improves anti-interference and robustness in complex environments.

The rest of the paper is organized as follows. In Section "The GE-WA model and pre-trained VGG-19 network", the proposed fusion method is introduced in detail. In Section "The proposed method implementation steps and strategies", we briefly summarize the image fusion process. In Section "Experiments and results analysis", experimental setup, results and analysis are showed. In Section "Conclusions", the conclusions of this paper are presented.

## The GE-WA model and pre-trained VGG-19 network

In order to obtain a fusion image that not only contains the temperature distribution of target and the details of the scene, but also suppresses the interfering light source and halo in the background. According to the principle of affine transformation^30^, visible and infrared images collected simultaneously in the same surveillance scene are registered, which the infrared image is used as the reference image. Because Laplacian transform can quickly enhance the detailed information of the image, find the edge and texture features of the image. Therefore, this paper uses Laplace sharpening to obtain the basic image and detail content of the image.

The registered source image $I_{k}$ is decomposed into basic image $I_{k}^{\text{b}}$ and detail content $I_{k}^{d}$ (*k* = 1, it means infrared image. *k* = 2, it means visible image.) using the Laplacian sharpening^31^, and different fusion strategies were used to fuse $I_{k}^{\text{b}}$ and $I_{k}^{d}$ respectively. The $I_{k}^{\text{b}}$ represents the approximate component of the image, which can reflect the contour characteristics of the image, and the $I_{k}^{d}$ represents the detail component of the image, which can express detailed information of the image and is also the most sensitive part of human eye recognition and machine vision^9^. Therefore, choosing the fusion rules of $I_{k}^{\text{b}}$ and $I_{k}^{d}$ is very important for the quality of fusion image.

**Figure 1:**
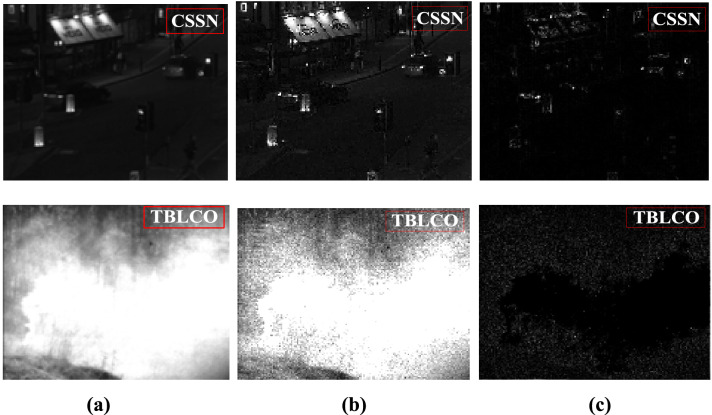
Visible image, basic image and detail image: (**a**) visible image, (**b**) basic image, (**c**) detail content.

The visible images of the complex street scene at night (CSSN) and the target of battlefield light curtain obscures (TBLCO) are sharpened by the Laplacian operator, the basic images and detailed contents are shown in Fig. 1.

### Basic image fusion strategy using GE-WA model

According to Fig. 1, the halo part of the visible image is concentrated in the basic image, that is, the basic image fusion strategy is to eliminate image halo as the main goal. Through a large number of experiments, in the basic image after Laplace sharpening, the gray of visible image at the halo part is close to a constant, and is significantly larger than the non-halo area, and the gray level changes show a Gaussian distribution. Because the Laplacian operator is a differential operator, it has the characteristics of enhancing the region of grayscale mutation and weakening the region of slowly changing grayscale.

Therefore, this paper designs a basic image fusion scheme based on the Gaussian Estimation-Weighted Average (GE-WA) model. The saliency coefficient of $I_{1}^{\text{b}}$ is automatically adjusted with the gray of $I_{2}^{\text{b}}$ in the proposed model, and the function of saliency coefficient is constructed as Eq. (1).

1 $$P_{{{\text{VIS}}}}^{b} \left( {x,y} \right) = \exp \left( {\frac{{{ - }\left( {I_{2}^{\text{b}} \left( {x,y} \right) - E} \right)^{2} }}{{\varepsilon \sigma^{2} }}} \right),$$

where $P_{{{\text{VIS}}}}^{\text{b}} \left( {x,y} \right)$ is the saliency coefficient of $I_{{2}}^{\text{b}}$ at coordinates (x, y). $I_{{2}}^{\text{b}} \left( {x,y} \right)$ is the gray of $I_{{2}}^{\text{b}}$ at coordinates (x, y). *E* is the halo constant after the Laplacian sharpening. According to the brightness of $I_{{2}}^{\text{b}}$, the mean of $I_{{2}}^{\text{b}}$ is used as the value of *E*. *ε* is the adjustment factor, which represents the intensity of change at the critical point of halo and non-halo of the image. $\sigma^{2}$ is the variance of $I_{{2}}^{\text{b}}$.

It can be seen from Eq. (1) that $P_{{{\text{VIS}}}}^{\text{b}}$ approaches 0 at the highlight and halo parts of $I_{{2}}^{\text{b}}$, $P_{{{\text{VIS}}}}^{\text{b}}$ gradually decreases with the drop of brightness $I_{{2}}^{\text{b}}$, and the $P_{{{\text{VIS}}}}^{\text{b}}$ is largest at the mean of $I_{{2}}^{\text{b}}$. Therefore, according to the change regular pattern of the saliency coefficient of $I_{k}^{\text{b}}$, the saliency map after eliminating the highlight and halo areas of $I_{{2}}^{\text{b}}$ is calculated by Eq. (2).

2 $$A_{{\text{VIS}}}^{\text{b}} \left( {x,y} \right) = \left( {{1 - }P_{{{\text{VIS}}}}^{\text{b}} \left( {x,y} \right)} \right)I_{1}^{\text{b}} \left( {x,y} \right) + P_{{{\text{VIS}}}}^{\text{b}} \left( {x,y} \right)I_{2}^{\text{b}} \left( {x,y} \right),$$

where $A_{{\text{VIS}}}^{\text{b}} \left( {x,y} \right)$ is the saliency map of $I_{{2}}^{\text{b}}$ at coordinates (x, y).

In the basic image fusion process, the highlight and halo parts of $I_{{2}}^{\text{b}}$ mainly selects the information of $I_{{1}}^{\text{b}}$, and weighted fusion is performed in the non-halo parts to obtain useful information of $I_{{1}}^{\text{b}}$ and $A_{{\text{VIS}}}^{\text{b}}$. $I_{{1}}^{\text{b}}$ and $A_{{\text{VIS}}}^{\text{b}}$ are weighted and fused by Eq. (3), and fusion basic image $F_{\text{b}}$ can be expressed as:

3 $$F_{b} \left( {x,y} \right) = \alpha_{1} I_{2}^{\text{b}} \left( {x,y} \right) + \alpha_{2} A_{VIS}^{b} \left( {x,y} \right),$$

where $F_{b} \left( {x,y} \right)$ is the gray of $F_{b}$ at coordinates (x, y). $\alpha_{1}$ and $\alpha_{2}$ are the weights of $I_{2}^{\text{b}}$ and $A_{VIS}^{b}$, respectively. In this paper, in order to preserve the background information as much as possible, $\alpha_{1} = I_{{2}}^{\text{b}} /\left( {I_{{2}}^{\text{b}} + A_{{\text{VIS}}}^{\text{b}} } \right)$, $\alpha_{2} = A_{{\text{VIS}}}^{\text{b}} /\left( {I_{2}^{\text{b}} + A_{{\text{VIS}}}^{\text{b}} } \right)$.

### Detail content fusion strategy based on pre-trained VGG-19 network

**Figure 2:**
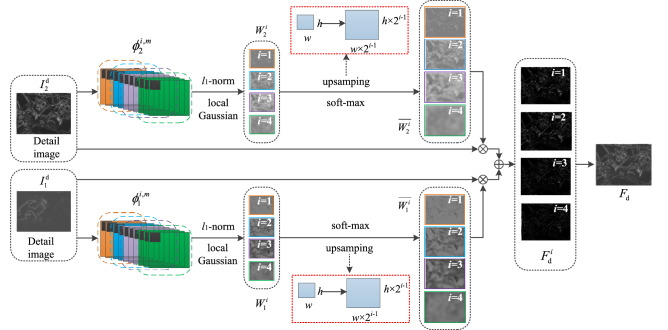
The fusion strategy framework of detailed images.

In recent years, image processing technology using deep learning has become one of the research hotspots in the field of visual images. Since VGG network is developed on the basis of the AlexNet network^32^, it has good generalization ability and can extract the depth features of each layer of the image^33,34^. Therefore, a new fusion strategy of detail content using the pre-trained VGG-19 network model is designed, and its fusion framework is shown in Fig. 2. First, the pre-trained VGG-19 network^35^ is used to extract the multi-layer depth features of the detail content $I_{k}^{d}$, then the weight map of depth feature is constructed from the multi-layer depth features, and finally the fused detail content $F_{d}$ is reconstructed according to the obtained weight map and $I_{k}^{d}$.

The depth feature $\phi_{k}^{i,m}$ in the *i-*th layer is obtained by the VGG-19 network model, and its expression is as follows

4 $$\phi_{k}^{i,m} = f_{i} \left( {I_{k}^{\text{d}} } \right),$$

where $f_{i} \left( \cdot \right)$ is the *i*-th layer of VGG-19 network. $\phi_{k}^{i,m}$ is the depth feature of $I_{k}^{\text{d}}$ in the *i-*th layer. *i* is the number of neural network layers, *i* ∈ {1,2,3,4}. *m* is the number of channels in the *i*-th layer, *m* ∈ {1,2,…,*M*}, *M* = 64 × 2i-1, *k* ∈ {1,2}.

Because *l*_1_-norm has the “sparse solution” characteristic of the regularization term, it is more suitable for feature screening, find out the “key” features, and sets the unimportant features to 0. According to Eq. (4), $\phi_{k}^{i,m}$ is an *M*-dimensional feature vector, that is, the *M*-dimensional feature vector of $\phi_{k}^{i,m}$ at coordinates (x, y) is represented by $\phi_{k}^{i,1:M} \left( {x,y} \right)$.

According to the feature vector $\phi_{k}^{i,1:M} \left( {x,y} \right)$ of different depths, *l*_1_-norm is used to calculate the rough saliency map $C_{k}^{i}$ of $I_{k}^{d}$. $C_{k}^{i}$ is calculated by Eq. (5).

5 $$C_{k}^{i} \left( {x,y} \right) = \left\| {\phi_{k}^{i,1:M} \left( {x,y} \right)} \right\|_{1} ,$$

where $C_{k}^{i}$ is the rough saliency map of $I_{k}^{d}$ in the *i*-th layer. $C_{k}^{i} \left( {x,y} \right)$ is the saliency of $C_{k}^{i}$ at the coordinates (x, y).

The fine saliency map $\overline{C}_{k}^{i}$ of $C_{k}^{i}$ is calculated by a regional Gaussian operator, which makes the fusion algorithm robust to registration error. $\overline{C}_{k}^{i}$ is calculated by Eq. (6).

6 $$\overline{C}_{k}^{i} \left( {x,y} \right) = \frac{{\sum {_{\beta = - r}^{r} \sum {_{\alpha = - r}^{r} w\left( {x + \alpha ,y + \beta } \right)C_{k}^{i} \left( {x + \alpha ,y + \beta } \right)} } }}{{\left( {2r + 1} \right)^{2} }},$$

where *w*(x, y) is the Gaussian operator at coordinates (x, y). The Gaussian operator uses a 2D Gaussian convolution kernel. *r* is the radius of the regional block. If *r* is larger, the fusion method has better robustness, but the more detailed information is lost, the area radius *r* = 1 in this paper.

The initial weight map $W_{k}^{i} \left( {x,y} \right)$ of $\overline{C}_{k}^{i}$ in the *i*-th layer is calculated by soft-max operator, and the calculation Eq. (7) is as follows.

7 $$W_{k}^{i} \left( {x,y} \right) = \frac{{\overline{C}_{k}^{i} \left( {x,y} \right)}}{{\sum {_{m = 1}^{K} \overline{C}_{m}^{i} \left( {x,y} \right)} }},$$

where *K* is the total number of $\overline{C}_{k}^{i}$, in this paper, *K* = 2. $W_{k}^{i} \left( {x,y} \right)$ is the value of $W_{k}^{i}$ at coordinates (x, y).

The pooling layer in the VGG network uses a sub-sampling method, that is, each pooling operation adjusts the size of the feature map to 1/*s* times the size of the source image, where *s* is the step size of the pooling layer. In the VGG-19 network, *s* = 2. Therefore, the size of the feature map corresponding to the depth of the *i-*th layer is 1/2^*i*-1^ times the size of the detail content (overlap pooling operation).

The inverse operation of the pooling process is adopted for $W_{k}^{i}$, and the size of $W_{k}^{i}$ is reconstructed by the up-sampling operator to make it the same size as $I_{k}^{d}$. Four pairs of weight maps $\overline{W}_{k}^{i}$ with different depths are obtained by Eq. (8).

8 $$\overline{W}_{k}^{i} = T\left( {W_{k}^{i} } \right),$$

where $T\left( \cdot \right)$ is the inverse operation of the pooling process by the up-sampling function. The size of $\overline{W}_{k}^{i}$ is (*M* + *p*)*(*N* + *q*), and the size of $W_{k}^{i}$ is M*N. $p,q \in \{ 0,1, \cdots ,2^{i - 1} - 1\}$.

According to the obtained four pairs of $\overline{W}_{k}^{i}$, the initial fusion detail image $F_{d}^{i}$ corresponding to each pair of weight maps is calculated by Eq. (9).

9 $$F_{d}^{i} \left( {x,y} \right) = \sum {_{n = 1}^{K} } \overline{W}_{n}^{i} \left( {x,y} \right) \cdot I_{n}^{d} \left( {x,y} \right),$$

The maximum selection strategy is adopted to select the maximum value of four groups $F_{d}^{i}$ at the same coordinate, and the fusion detail content $F_{\text{d}}$ is obtained through Eq. (10).

10 $$F_{d} \left( {x,y} \right) = \max \left[ {F_{d}^{i} \left( {x,y} \right)|i \in \left\{ {1,2,3,4} \right\}} \right],$$

### Refactoring

The fusion basic image *F*_b_ and detail content *F*_d_ are reconstructed by Eq. (11), and the final fusion image *F* is obtained.

11 $$F\left( {x,y} \right) = F_{b} \left( {x,y} \right) + F_{d} \left( {x,y} \right),$$

where $F\left( {x,y} \right)$ is the pixel value of the fusion image *F* at coordinates (x, y).

## The proposed method implementation steps and strategies

The main steps of the visible and infrared images fusion method based on GE-WA model and deep learning framework was proposed are as follows:

**Step 1**: Perform Laplacian sharpening on the source image $I_{k}$ after registration to obtain the basic image $I_{k}^{\text{b}}$ and the detail content $I_{k}^{d}$.

**Step 2**: According to the gray distribution rule of $I_{k}^{\text{b}}$, design the Gaussian estimation model $P_{{{\text{VIS}}}}^{b}$ to obtain the significant coefficients of $I_{k}^{\text{b}}$.

**Step 3**: Construct a basic image fusion strategy based on the GE-WA model, perform halo processing and weighted fusion on $I_{k}^{\text{b}}$ to obtain the fusion basic image $F_{\text{b}}$.

**Step 4**: Use the pre-trained VGG-19 network model to obtain the multi-layer depth features of $I_{k}^{d}$, utilize *l*_1_-norm to calculate the 4-layer rough saliency maps $C_{k}^{i}$ of $I_{k}^{d}$, and calculate the fine saliency maps $\overline{C}_{k}^{i}$ of each layer $C_{k}^{i}$ based on the regional Gaussian operator.

**Step 5**: Calculate the initial weight map $W_{k}^{i}$ of $\overline{C}_{k}^{i}$ in the *i*-th layer by the soft-max operator, and use the pooling inverse operation and up-sampling operator to reconstruct the size of $W_{k}^{i}$ to obtain the weight map $\overline{W}_{k}^{i}$ of the *i-*th layer.

**Step 6**: Repeat Step5, obtain 4 pairs of weight maps $\overline{W}_{k}^{i}$ in turn, and use weighted fusion to obtain the initial fusion detail content $F_{d}^{i}$, and use the maximum selection strategy to obtain the maximum value of the 4 layers $F_{d}^{i}$ at the same coordinate, and then obtain the fusion detail content $F_{\text{d}}$.

**Step 7**: Reconstruct the fusion basic image $F_{\text{b}}$ and fusion detail content $F_{\text{d}}$ to obtain the fusion image *F*.

**Figure 3:**
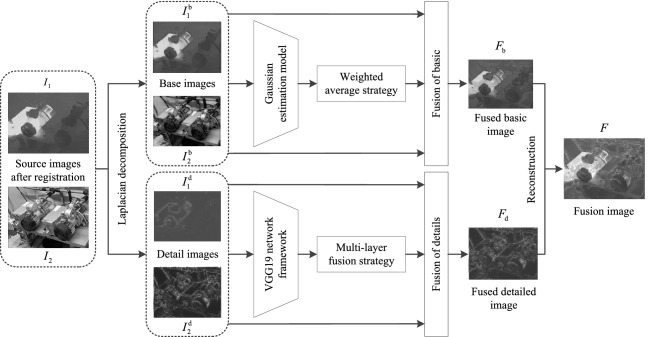
The overall framework diagram of the proposed method.

The framework of the fusion method is shown in Figure 3.

## Experiments and results analysis

In order to verify the effectiveness and advancement of the proposed method in different complex lighting environments, a test dataset containing halo images is constructed, and the source image that has strictly registration is experimentally verified. At the same time, the proposed algorithm will be compared with the results of other seven typical fusion methods (WLS^36^, LatLRR^37^, NSCT-Mean-AE^38^, NSCT-Edge-PCNN^26^, JSRSD^39^, DLF^40^, ResNet-ZCA^17^), and the experimental results will be discussed in terms of subjective visual effects and objective evaluation indicators.

All the experiments are implemented in the Matlab R2020a software platform, the CPU is 3.20GHz Intel(R) Core(TM), the RAM is 16.00GB. In the experiment, the parameter settings of the seven typical fusion algorithms are consistent with the original literature.

### Subjective visual analysis

Four types of typical source images are selected from the test dataset in this paper for fusion experiments, which are Electrical Equipment Fault Diagnosis (EEFD), TBLCO, CSSN, and Road Car Halo Effect (RCHE).

1. Experiment 1: Perform a fusion test on an EEFD source image with a resolution of 305×229. The experimental results are shown in Fig. 4.

**Figure 4:**
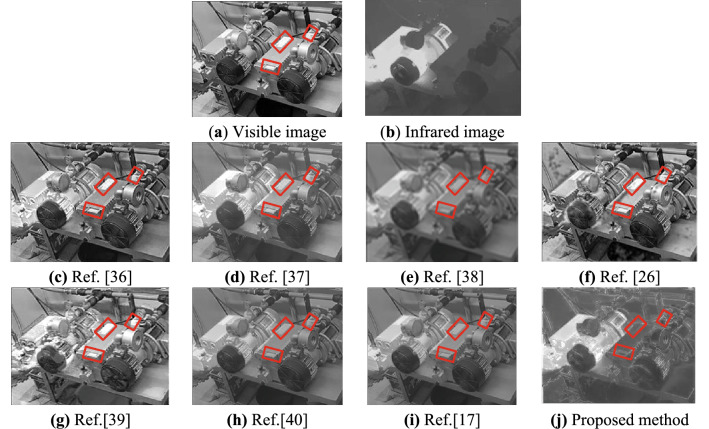
Visible and infrared images (EEFD) and their fusion results.
2. Experiment 2: Perform a fusion test on a TBLCO source image with a resolution of 768 × 576. The experimental results are shown in Fig. 5.

**Figure 5:**
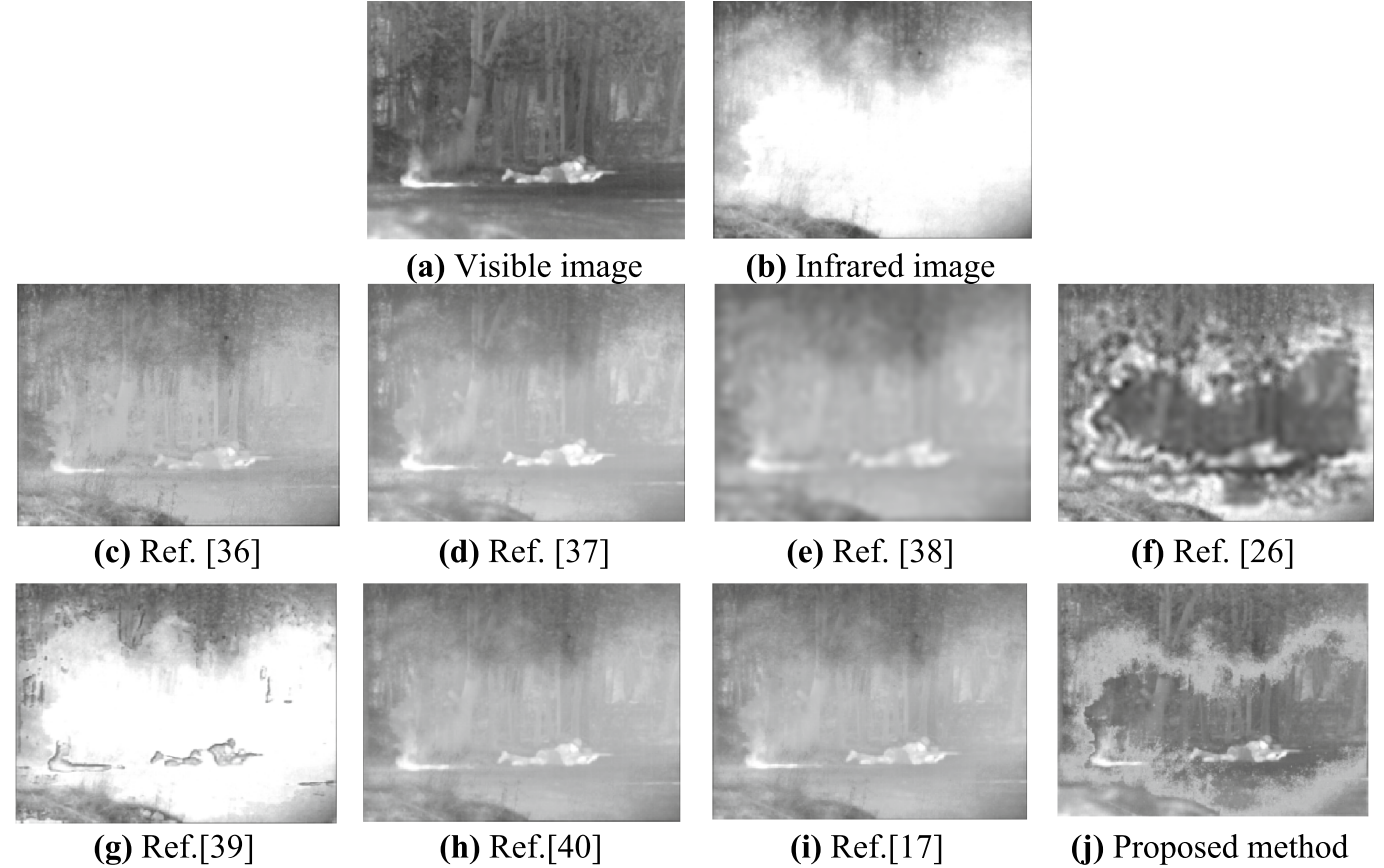
Visible and infrared images (TBLCO) and their fusion results.
3. Experiment 3: Perform a fusion test on a CSSN source image with a resolution of 632 × 496. The experimental results are shown in Fig. 6.

**Figure 6:**
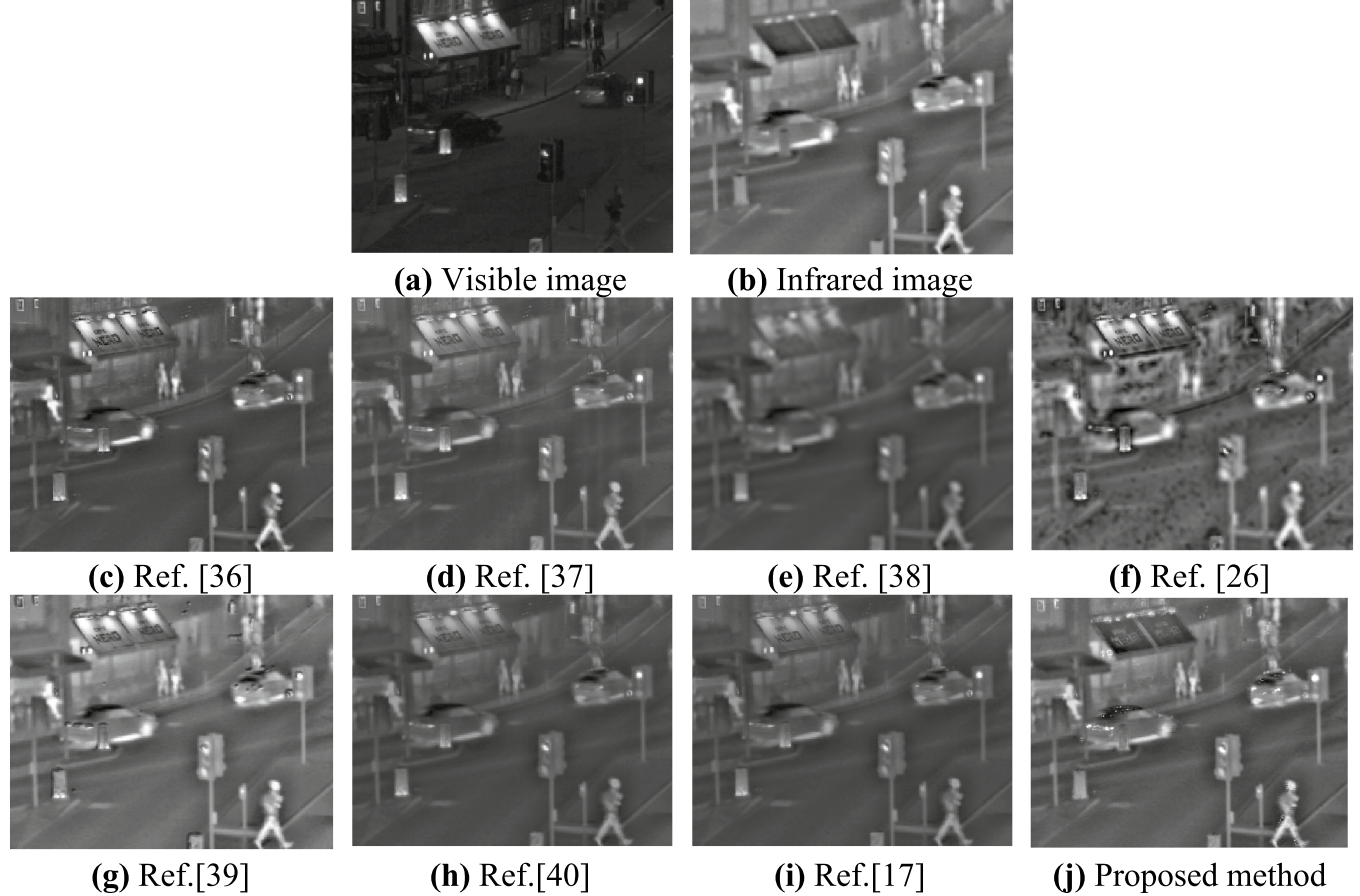
Visible and infrared images (CSSN) and their fusion results.
4. Experiment 4: Perform a fusion test on a RCHE source image with a resolution of 348 × 260, and the experimental results are shown in Fig. 7.

**Figure 7:**
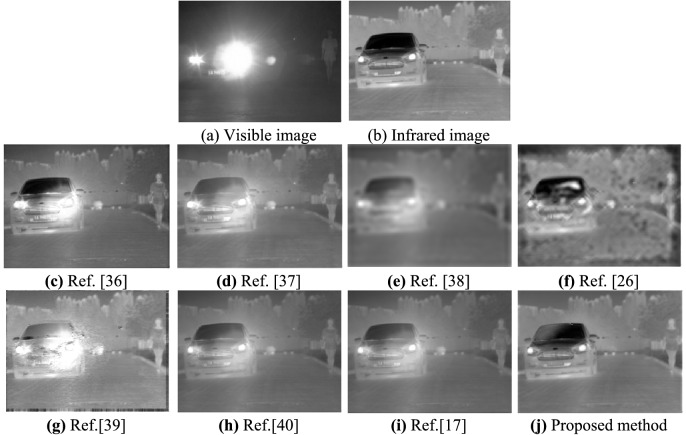
Visible and infrared images (RCHE) and their fusion results.

By comparing the fusion results of each algorithm in the four types of scenes (as shown in Figs. 4, 5, 6 and 7), it can be seen that the fusion image fused by the proposed method has rich detailed information, significant targets, and good visual effects. All the comparison methods have different fusion defects, as shown in Fig. 4c, d, f, g, the interference target in the red marked box cannot be suppressed. The details of the image are reduced in Fig. 4e, h, i, the image background details and target information are blurry in Fig. 5c–e, h, i, the halo part appears distortion in Fig. 5f, g. There are obvious shadow areas in Fig. 6e and f, the halo parts can’t be eliminated in Fig. 6c and g, the outline of the target is blurred and part of the detailed information is lost in Fig. 6d, h, i. The halo part can’t be eliminated in Fig. 7c, d, g, the image are less details and is distorted in Fig. 7e–g.

In summary, the fusion image using the Ref.^36^ can better retain the target and improve the overall contrast, but can’t eliminate the halo phenomenon in the image. The fusion image using the Refs.^38^ and Ref.^26^ has serious distortion, fewer texture features, and blurry edges. The fusion image using the Refs.^37^,^40^ and Ref.^17^ has the problems that the overall image is dark, the details of the object are blurred, and the visual effect is not good. The fusion image using the Ref.^39^ has distortion in the halo part, and the fusion result is not clear. The proposed method can completely eliminate the halo of visible image, and the object in the fusion image is clear and the edge is obvious, which is more in line with human visual characteristics.

### Objective indicators analysis

In order to more objectively evaluate the fusion performance and halo elimination effect of the proposed method and the comparison methods in different environments, the paper selects five typical indicators to analyze the fusion images of the above seven comparison methods and the proposed method. In addition, the average running time (ART) is used as an important indicator to evaluate the operating efficiency of different fusion methods. The ART of the algorithm described in Ref.^39^ can’t be reproduced, so that the actual running time is especially used.

(1) Root Mean Square Error (RMSE). RMSE represents the global size of the pixel error between the fusion image and the reference image, which is used to measure the quality of the image^41^. The larger the value of RMSE, the better the effect of image halo elimination. The value $R_{{\text{RMSE}}}$ of RMSE is obtained by Eq. (12).

12 $$R_{{\text{RMSE}}} = \frac{1}{2}\sum\limits_{k = 1}^{2} {\sqrt {\frac{1}{M \times N}\sum\limits_{x = 1}^{M} {\sum\limits_{y = 1}^{N} {\left[ {F\left( {x,y} \right){ - }I_{k} \left( {x,y} \right)} \right]^{2} } } } } ,$$

where *M* × *N* is the image resolution.

(2) Average Gradient (AG). AG is used to describe the overall spatial activity of the image^42^, reflecting the small detail contrast and texture change ability in the image. The larger the AG value is, the clearer the fused image is. The value $R_{{\text{AG}}}$ of AG is obtained by Eq. (13).

13 $$R_{{\text{AG}}} = \frac{1}{2}\sum\limits_{k = 1}^{2} {\frac{1}{M \times N}\sum\limits_{x = 1}^{M} {\sum\limits_{y = 1}^{N} {\sqrt {\nabla f_{x}^{2} \left( {x,y} \right) + \nabla f_{y}^{2} \left( {x,y} \right)} } } } ,$$

where ∇*f*_*x*_ and ∇*f*_*y*_ are the first-order difference operators of the image in the horizontal and vertical directions, respectively.

(3) Halation Elimination (HE). HE is used to evaluate the ability of the fusion image to eliminate the interference of halo part, and can more objectively evaluate the subjective effect of the human eye^9^. The larger the value of HE, the better the effect of the fusion method on eliminating halo in visible images. The value $R_{{{\text{HE}}}}$ of HE is obtained by Eq. (14).

14 $$R_{SSIM} = SSIM\left( {F,I_{1} } \right) + \left( {1 - SSIM\left( {F,I_{2} } \right)} \right),$$

where $R_{SSIM}$ is the index value of structural similarity. $SSIM( \cdot )$ is the operator of structural similarity^43^.

4. Deviation Index (DI). DI represents the relative deviation between the fusion image and the reference image. The smaller the DI value is, the smaller the relative difference between the two in spectral information^41^, that is, the smaller the effect of the fused image on the halo elimination of the reference image. The value $R_{DI}$ of DI is obtained by Eq. (15).
  15 $$R_{DI} = \frac{1}{2}\sum\limits_{{{\text{k = }}1}}^{2} {\frac{1}{M \times N}\sum\limits_{x = 1}^{M} {\sum\limits_{y = 1}^{N} {\frac{{\left| {F\left( {x,y} \right){ - }I_{k} \left( {x,y} \right)} \right|}}{{F\left( {x,y} \right)}}} } } ,$$
5. Fusion Quality (FQ). FQ is used to characterize the overall fusion performance of the infrared and visible image fusion methods. The larger the value of FQ is, the better the performance of the fusion method. The value $R_{{{\text{FQ}}}}$ of FQ is obtained by Eq. (16)^43^.
  16 $$R_{{{\text{FQ}}}} { = }\sum\limits_{i = 1}^{p} {w_{i} y_{i} , \, y_{i} = \frac{{x_{j} - x_{\min } }}{{x_{\max } - x_{\min } }}} ,$$
  where $w_{i}$ is the weight of the indicator *i*. *p* is the total number of indicators, *p* = 4. $y_{i}$ is the normalized value of index *i*. $x_{\max }$ is the maximum value of the indicator *i* of all methods. $x_{\min }$ is the minimum value of the indicator *i* of all methods. $x_{j}$ is the value of the indicator *j* of different method. *i* = 1, 2, 3, 4, representing RMSE, AG, HE, and DI indicators, respectively.

The objective evaluation indicator values of the fusion images corresponding to the four typical source images are obtained by Eqs. (12–16) and ART, as shown in Table 1.

**Table 1:** Performance indicators of different algorithms under four types of source images.

Images	Indicators	Methods							
		Ref.^36^	Ref.^37^	Ref.^38^	Ref.^26^	Ref.^39^	Ref.^40^	Ref.^17^	Proposed method
EEFD	RMSE	0.1486	0.1760	0.1534	0.1456	0.1696	0.1533	0.1480	0.1763
	AG	0.0147	0.0052	0.0006	0.0142	0.0138	0.0047	0.0039	0.0051
	HE	1.2641	0.8946	0.9889	0.3724	0.5638	0.8698	0.8668	1.5403
	DI	0.6091	0.2462	0.2858	0.5099	0.3502	0.2805	0.2826	0.2868
	FQ	2.8621	1.7685	0.8926	1.6935	2.1716	1.0642	0.8368	2.4303
	ART	0.8810	11.3264	18.1150	61.8644	52.9837	3.7807	1.3801	1.1387
TBLCO	RMSE	0.2770	0.2970	0.3163	0.3224	0.2950	0.2771	0.2783	0.3236
	AG	0.0024	0.0009	0.0001	0.0025	0.0044	0.0008	0.0007	0.0015
	HE	1.2855	0.6493	0.5342	1.0815	0.1896	0.5445	0.5540	1.3296
	DI	1.1600	0.4125	0.4301	1.0715	0.4074	0.4303	0.4306	0.5821
	FQ	2.4953	1.0154	1.1760	3.1845	1.3868	0.5086	0.5194	2.5592
	ART	3.0534	118.9385	239.1666	854.1377	370.4767	9.9570	5.3957	7.0884
CSSN	RMSE	0.1499	0.1665	0.1460	0.1525	0.1488	0.1440	0.1437	0.1858
	AG	0.0025	0.0012	0.0001	0.0020	0.0022	0.0008	0.0007	0.0162
	HE	0.8071	1.0429	1.1283	1.3041	1.4056	1.0685	1.0590	1.6951
	DI	0.6201	0.5378	0.6085	0.5746	0.4109	0.6077	0.6063	0.4900
	FQ	1.2970	1.4827	1.3596	1.6672	0.9269	1.2846	1.2532	3.3779
	ART	2.2458	74.5470	127.6953	440.1966	538.3648	7.1347	4.5216	5.0178
RCHE	RMSE	0.1643	0.2108	0.1983	0.1702	0.1758	0.1884	0.1857	0.1830
	AG	0.0045	0.0031	0.0001	0.0038	0.0037	0.0025	0.0024	0.0024
	HE	1.2302	0.6476	0.7663	1.4553	0.5697	0.7839	0.7917	1.8361
	DI	0.3905	0.2850	0.3168	0.7548	0.2827	0.3116	0.3119	0.7656
	FQ	1.7448	1.7511	0.9563	2.6600	1.0776	1.2997	1.2161	2.9307
	ART	0.3497	16.6214	36.9075	126.7217	70.3039	3.8821	1.4798	1.5973

In the experiment, 20 sets of source images in the database were randomly selected for fusion, and six typical objective evaluation indicators were obtained. At the same time, the average value of each type of objective evaluation indicator is calculated by combining the data of the fused image in Table 1, as shown in Table 2.

**Table 2:** Average performance indicators of different fusion methods.

Methods	Indicators					
	RMSE	AG	HE	DI	ART	FQ
Ref.^36^	0.1748	0.0069	1.3753	0.6298	**1.6629**	2.0639
Ref.^37^	0.1786	0.0027	0.5961	*0.3128*	58.5104	0.3993
Ref.^38^	0.1789	*0.0002*	0.6506	0.3427	105.7410	0.4064
Ref.^26^	0.1806	0.0062	0.5606	**0.7812**	*367.2269*	1.5638
Ref.^39^	0.1785	0.0072	*0.4606*	0.4120	218.4803	0.6901
Ref.^40^	*0.1683*	0.0022	0.6006	0.3372	6.9420	0.3002
Ref.^17^	0.1695	0.0020	0.5922	0.3371	3.4575	*0.2927*
Proposed method	**0.2435**	**0.0205**	**1.3999**	0.4397	4.2888	**3.2708**

Significant values are in [bold and italics].

**Figure 8:**
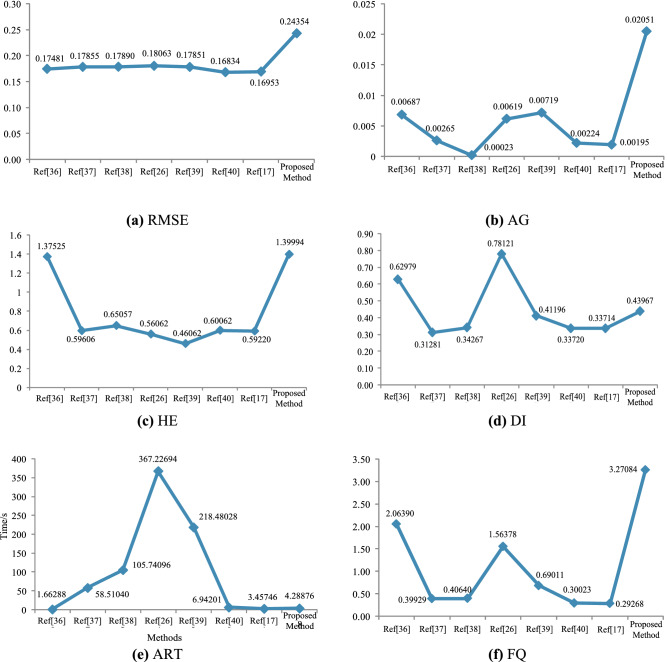
Broken-line graphs of evaluation indicators.

According to the data in Table 2, the broken-line graphs of different evaluation indicators are drawn (as shown in Fig. 8), and the proposed method and the seven comparison methods are objectively analysis.

According to the four typical evaluation indicators, FQ and ART in Table 2 and Fig. 8, the RMSE, AG, and HE values of the proposed method are higher, indicating that the proposed method has higher fusion quality than other comparison methods, and obvious advantages in eliminating halo in the visible image, which is consistent with the subjective visual analysis. The DI value of the Ref.^37^, Ref.^40^ and Ref.^17^ are relatively small, indicating that the three image fusion methods are prone to losing the details of visible and infrared images. The HE value of the Ref.^39^ is small, which means that this method is not suitable for image fusion under complex lighting environment. The AG value of the Ref.^38^ is the smallest, which means that the fusion image obtained by this method has low overall clarity and poor visual effect. The ART value of Ref.^38^, Ref.^26^ and Ref.^39^ is larger, namely, the fusion method of multi-scale decomposition is easy to reduce the fusion efficiency. The ART value of the proposed method is smaller, which means that the proposed method is more suitable for fields with high real-time requirements.

The above analysis shows that the RMSE, AG and HE values of the proposed method are the largest, indicating that the image fusion quality of the proposed method is better than other comparison methods. The DI value of the proposed method is relatively small, which is due to the fact that the fusion image and the source image have a certain degree of distortion when eliminating the halo of the visible image. In addition, the comprehensive evaluation FQ value of the proposed method is the largest, indicating that the proposed method can obtain more detailed information of the fusion image, higher definition and stronger anti-interference ability.

## Conclusions

The proposed method uses Laplacian sharpening to realize the rapid separation of general and detailed features in the source image, and obtains the basic image containing halo and contour and the detail content containing the texture and edge features. The basic image fusion strategy based on the GE-WA model is adopted to realize the reliable fusion of the general features of the visible and infrared images, eliminate the halo parts in the visible image, and reduce the redundancy of the background information in the fused image. The pre-trained VGG-19 network and the multi-layer fusion strategy are used to realize the fusion of different depth features of the visible and infrared images, and obtain the detail content after fusion. The fusion image is reconstructed by the basic image and detail content after fusion.

The experimental results show that the performance of the proposed method is similar to the existing methods, and it is better than the comparison methods in eliminating halo. The proposed method using the GE-WA model and VGG-19 network overcomes the shortcomings of traditional methods that can’t extract image depth and detail information, can obtain more comprehensive, reliable and rich scene information, and can achieve rapid image fusion in a variety of different scenarios.

The RMSE, AG and HE values of the proposed method are the largest, and the ART value is smaller, indicating that the image fusion quality and fusion efficiency of the proposed method are better than those of the comparison methods. In order to eliminate the halo parts in the visible image, the proposed method distorts the structural information of the fused image and the visible image to a certain extent, resulting in the decrease of the DI value. However, the comprehensive evaluation FQ value of the proposed method is the largest, indicating that the proposed method is more suitable for visible and infrared image fusion in complex environments. In the future, the team will further study more effective multispectral image fusion methods and the corresponding fusion image evaluation indicators.

## Data Availability

Infrared and visible image/video datasets can be available from the following URLs: https://figshare.com/articles/dataset/TNO_Image_Fusion_Dataset/1008029. https://www.ino.ca/en/technologies/video-analytics-dataset/. The other data used to support the findings of this study are available from the corresponding author upon request.
